# Changes in antioxidant capacity and gut microbiota in mice after intake of camel milk

**DOI:** 10.3389/fcimb.2025.1621031

**Published:** 2025-09-03

**Authors:** Jianwen Wang, Wanlu Ren, Shibo Liu, Zexu Li, Yaqi Zeng, Jun Meng, Xinkui Yao

**Affiliations:** ^1^ College of Animal Science, Xinjiang Agricultural University, Urumqi, China; ^2^ Xinjiang Key Laboratory of Equine Breeding and Exercise Physiology, Urumqi, China

**Keywords:** gut microbiota, antioxidant capacity, camel milk, metagenomic, mice

## Abstract

Fermented camel milk offers significant nutritional benefits, enriched with probiotics that generate bioactive compounds advantageous to human health. In order to investigate the effects of camel milk with different treatments on Antioxidant Capacity and Gut Microbiota in mice, 32 ICR mice were selected and randomly divided into 4 groups, including gavage with 10 mL/kg body weight of distilled water (DW Group), camel milk (CM Group), fermented camel milk (FCM Group), and pasteurized fermented camel milk (PFCM Group) every morning, respectively. After 28 days, liver and colon samples were collected to assess liver antioxidant capacity, and metagenomic analysis was performed on alterations in microbial community structures. Results demonstrated that all camel milk treatments elevated liver total protein levels while reducing MDA and SOD activity. In addition, the PFCM group had the highest total antioxidant capacity and the lowest SOD content. In addition, the intestinal microorganisms of mice changed at the phylum, genus and species levels after being gavaged with camel milk of different treatments. A total of 4732 microorganisms were identified, of which 259, 222, 116 and 164 were unique to the DW, CM, FCM and PFCM groups, respectively. The relative abundances of Adlercreutzia caecimuris, Adlercreutzia mucosicola and Enterorhabdus sp. P55 were significantly higher in the CM, FCM and PFCM groups than in the DW group, and the relative abundances of Parvibacter caecicola, Adlercreutzia muris and Roseburia sp. 1XD42-69 were significantly higher in the CM and PFCM groups than in the DW group. In addition, the relative abundances of Faecalibaculum rodentium, Alistipes muris and Limosilactobacillus reuteri were different between the CM and FCM groups. The results of the correlation analysis between the relative abundance of microbial species and antioxidant indices showed that Adlercreutzia mucosicola, Adlercreutzia muris, Lactobacillus acidophilus, and Enterorhabdus sp. P55 were significantly correlated with the antioxidant indices of mice. Further functional annotations indicated that these microorganisms might modulate antioxidant activity via metabolic and organismal systems. In summary, camel milk and fermented camel milk can play a positive role in regulating the intestinal flora of mice, thereby regulating the antioxidant capacity of mice and alleviating the effects of oxidative stress on the body. This study provides a scientific foundation for the further exploration and utilization of camel milk.

## Graphical Abstract

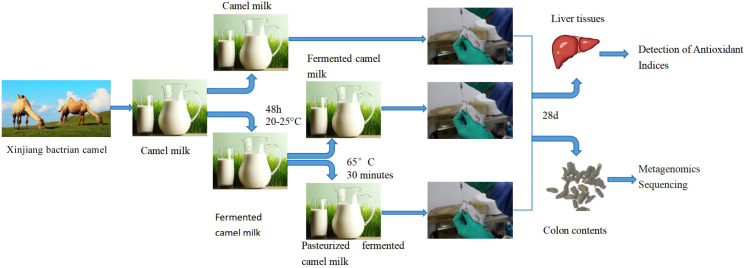

## 1 Introduction

As an important “invisible organ” of the human body, the intestinal microbiota plays multiple roles in maintaining the health of the host, among which the antioxidant function has attracted much attention (Shen et al., 2025). The intestinal flora helps to scavenge free radicals and reduce oxidative stress by directly secreting antioxidant substances (such as glutathione and superoxide dismutase) (Meng et al., 2022; Liu M. et al., 2024), and regulating the expression of host antioxidant enzymes (such as the Nrf2 pathway) (Niu et al., 2024). Dairy products are rich in probiotics and prebiotics, which play an important role in regulating intestinal microorganisms and antioxidant capacity. The live bacteria in fermented dairy products can colonize in the intestines, increase the abundance of beneficial bacteria such as bifidobacteria and lactic acid bacteria (Calatayud et al., 2021), and protect the integrity of the intestinal barrier by inhibiting lipid peroxidation and reducing malondialdehyde (MDA) levels (Wang et al., 2019), thereby preventing inflammation and metabolic diseases. In addition, ingredients such as lactoferrin (Bellés et al., 2023) and conjugated linoleic acid (CLA) (Deveci et al., 2023) in dairy products can inhibit the growth of pathogenic bacteria, promote the production of short-chain fatty acids (SCFA, such as butyrate), reduce the accumulation of reactive oxygen species (ROS), and exert antioxidant effects (He and Dong, 2023). Different dairy products have different nutritional and microbial compositions due to different livestock species, processing technology, etc., and the regulation of the antioxidant capacity will be different.

Camel milk is a nutrient-dense liquid, widely regarded as a vital food source in arid and semi-arid regions (Guo et al., 2024) and often referred to as “white desert gold”. Its nutritional profile closely resembles that of human milk, offering superior nutritional value compared to cow’s milk (Kocyigit et al., 2024). Beyond its role as a food product, camel milk has demonstrated therapeutic potential for a range of chronic diseases, owing to its potent antioxidant, immune-modulating, anti-inflammatory, and anti-apoptotic (Ahamad et al., 2017; Aqib et al., 2019). This may be related to the chemical composition of camel milk, especially the proteins, peptides and fatty acids in it. Studies have shown that the protein composition and properties in camel milk are quite different from those in cow milk, such as casein accounting for 53%-86% of the total protein, and whey protein accounting for 20%-25% of the total protein (Marete et al., 2024). The ratio of the four main caseins: α1-casein, αs2-casein, β-casein and κ-casein is 26:4:67:3, while the ratio in cow milk is 38:10:36:12 (Jiang et al., 2025). In addition, the whey protein in camel milk is mainly α-lactalbumin and serum protein, and does not contain β-lactoglobulin in cow milk that can cause allergies (Zhu et al., 2024). The α-lactalbumin in camel milk has a higher antioxidant potential than cow milk (Redwan and Uversky, 2022). The lactoferrin content in camel milk is 0.18-2.48 mg/ml, which is much higher than that in ruminant milk, which may also be the reason why camel milk has antioxidant capacity (Behrouz et al., 2022). In addition, the concentration of polar lipids or phospholipids (such as phosphatidylserine, phosphatidylethanolamine, phosphatidylinositol and sphingomyelin) in camel milk is much higher than that in other animal milks (goat milk and cow milk), which may also affect the antioxidant properties of camel milk (Benmeziane-Derradji, 2021). The high vitamin C content in camel milk (3-5 times higher than that in cow milk) is also an important reason for its antioxidant activity (Behrouz et al., 2022).

Dairy products derived from camel milk are primarily fermented, including sour camel milk, pasteurized camel milk, cheese, and ice cream, which are available in select markets (Ayyash et al., 2018). Additionally, the nutritional characteristics of fermented camel milk products have been documented (Ayyash et al., 2021). The study found that the microorganisms in fermented camel milk are mainly lactic acid bacteria, lactococci, leuconostoc and enterococci at the genus level, and the probiotics among them have the function of improving β cells (Bilal et al., 2024). The four probiotics isolated from fermented camel milk can regulate the CD4+/CD8+ cell ratio and thus alleviate the body’s immune deficiency (He et al., 2023). In addition, the bioactive peptides isolated from camel milk can also stimulate the expression of IFN-γ in mouse lymphocytes to play a role (Wang et al., 2023).

In summary, camel milk and fermented camel milk have important physiological functions.Few studies have explored the potential link between enhanced antioxidant capacity through various camel milk treatments and microbial modulation. These studies mainly focused on the microbial composition and function of fermented camel milk (Bilal et al., 2024; Zhadyra et al., 2025), as well as the regulation of fresh camel milk (Ali et al., 2024) on mouse intestinal microorganisms. This study hypothesizes that camel milk may elevate antioxidant capacity by modifying gut microbiota composition. To investigate this, the liver antioxidant capacity and gut microbiota of mice were assessed following gavage with camel milk subjected to different treatments. The objective was to determine whether camel milk can enhance antioxidant capacity through alterations in gut microbiota structure, offering novel perspectives for future research on the functional properties of camel milk.

## 2 Materials and methods

### 2.1 Experimental materials

The camel milk used in this study was sourced from Xinjiang Tarim bactrian camels, Tacheng, Xinjiang. Four camel milks were obtained by hand-milking in July, the third month after calving, and subsequently pooled. They were 5 years old and weighed (445.25 ± 7.71) kg at the time of milking. All camel milk was collected on the same day. All camel milk was divided into 3 portions, each weighing 500 g. One portion was directly stored at -20°C(camel milk). The remaining two portions were mixed with the remaining sour camel milk (1:1, v/v) and fermented at ambient temperature (20-25°C) for 48 hours. After fermentation, the fermented camel milk was divided into 2 portions, one of which was fermented camel milk and the other was pasteurized (65°C for 30 minutes) for pasteurized fermented camel milk. After the treatment, the fermented camel milk and pasteurized fermented camel milk were stored at -20°C. The pH of fermented camel milk is 4.8. The nutritional composition of all processed camel milk samples was presented in **Supplementary Tables S1**, **S2**. Thirty-two ICR mice (SPF grade), including 16 male mice and 16 female mice, 4 weeks old and weighing between 18-20 g, were acquired from the Animal Experiment Center of Xinjiang Medical University.

### 2.2 Experimental methods

#### 2.2.1 Experimental groups

Thirty-two ICR mice (18-20 g), evenly distributed by sex. Before the experiment, we numbered all mice according to their gender and then randomly divided them into four groups using a random number table: distilled water (DW), fresh camel milk (CM), fermented camel milk (FCM), and pasteurized fermented camel milk (PFCM), with four mice per cage, separated by sex. Each group consisted of one cage of male and one cage of female mice. All mice were born on the same day and maintained in the same manner including diet before the study. After one week of acclimation, during which they had unrestricted access to water and standard feed, the mice were gavaged based on body weight. All mice were maintained at a temperature of 22 ± 2°C with a 12 h light/dark cycle. Each group received 10 mL/kg body weight of the respective camel milk treatment daily, while the DW group was administered 10 mL/kg body weight of distilled water. Mouse weights were recorded weekly to adjust gavage volumes as needed. The weight data were found in **Supplementary Table S3**. Gavage was performed once daily for four consecutive weeks, with ad libitum access to food and water at all other times.

#### 2.2.2 Sample collection

On day 29 post-initial treatment, mice were killed by cervical dislocation following anesthesia with pentobarbital. Within 5 minutes after the mice were sacrificed, we collected liver tissues and colon contents from all 32 mice, with approximately 0.5 g of liver and 0.2 g of colon contents collected from each mouse. Liver tissue was immediately harvested (within 5 min), rinsed with saline, and used for antioxidant assay. The collected colonic contents were transferred to sterile, enzyme-free 10 mL centrifuge tubes, rapidly frozen in liquid nitrogen, and subsequently shipped to Novogene Company (Beijing, China) on drikold for metagenomic sequencing.

#### 2.2.3 Detection of antioxidant indices

Total protein (TP) concentrations in the liver were quantified using the BCA method (P0011, Beyotime Biotechnology, Shanghai, China). Catalase activity (CAT) (ADS-W-KY002, AIDISHENG, Yancheng, China), malondialdehyde (MDA) content (ADS-W-YH002, AIDISHENG, Yancheng, China), superoxide dismutase (SOD) activity (ADS-W-KY011-196, AIDISHENG, Yancheng, China), glutathione peroxidase (GSH-PX) activity (ADS067TE0, AIDISHENG, Yancheng, China), and total antioxidant capacity (TAC) (ADS003TO0, AIDISHENG, Yancheng, China) were measured using commercially available kits, following the manufacturer’s protocols. In simple terms, the BCA protein concentration detection method is to first prepare a protein standard with a concentration of 0.5 mg/ml and prepare a BCA working solution, then use an enzyme reader to measure the absorbance of samples with different protein standard concentrations at 562 nm, and calculate the functional relationship between protein concentration and absorbance. The liver tissue is added to 10 times the volume of pre-cooled RIPA lysis buffer containing 1× protease inhibitors and homogenized. After standing on ice for 30 minutes, centrifuge at 12,000 rpm for 30 minutes at 4°C, and the supernatant is collected as the prepared liver tissue test sample. After the test sample is diluted 60 times, 10 times the volume of BCA working solution is added. After standing at 37°C for 30 minutes, the absorbance at 562 nm is detected by an enzyme reader, and the protein concentration of the liver is calculated based on the functional relationship between protein concentration and absorbance.The preparation method of CAT, MDA, SOD, GSH-PX and TAC test samples is to add 10 times the volume of the extract to the liver tissue, homogenize it in an ice bath, and then centrifuge it at 4°C, and take the supernatant as the sample to be tested. Then, use an ELISA reader to detect the absorbance at 510nm (CAT), 532nm and 600nm (MDA), 560nm (SOD), 340nm (GSH-PX), and 593nm (TAC) according to the instructions, and add the corresponding test solution during the detection. Calculate CAT, MDA, SOD, GSH-PX, and TAC in liver tissue according to the formula provided in the instructions.

#### 2.2.4 Library construction and sequencing

DNA purity and integrity were assessed via agarose gel electrophoresis(AGE), followed by precise quantification of DNA concentration using Qubit. A Covaris ultrasonic device fragmented the DNA into approximately 350 bp segments. Library preparation involved end repair, A-tailing, adapter ligation, purification, and PCR amplification. Post-construction, the library was initially quantified using Qubit 2.0 and diluted to 2 ng/µL. The insert size was evaluated with the Agilent 2100, and the library’s effective concentration was determined through Q-PCR (effective concentration > 3nM) to confirm quality. Qualified samples underwent sequencing on the Illumina PE150 platform. The detection results of each mouse fecal microorganism ranged from 1.72G to 2.67G, and the obtained Raw Data ranged from 5,981 to 6,758.

### 2.3 Data analysis

#### 2.3.1 Intestinal microbial community analysis

The raw data were subjected to quality control and host filtering to yield clean data. Metagenomic assembly was performed on the clean data, followed by gene prediction. Gene prediction was conducted using MetaGeneMark(http://topaz.gatech.edu/GeneMark/), starting from the scaftigs assembled from individual samples. The resulting gene predictions for each sample were consolidated to eliminate redundancy, thereby constructing a comprehensive gene catalogue. The unigenes were compared against the NR database and the KEGG database via DIAMOND(https://github.com/bbuchfink/diamond/), and annotation was carried out using sequence alignments. Species classification and annotation were performed using the LCA algorithm in MEGAN 6. Microbial alpha diversity was assessed from the test results. A hypothesis test based on species abundance data across groups was performed using the MetaStat method to calculate p-values. The q-value was derived by adjusting the p-value, and species exhibiting significant differences were identified with a threshold of *q*<0.05. Differentially expressed functions across groups were analyzed using LEfSe (http://galaxy.biobakery.org/), with an LDA score > 2 indicating significant differences.

#### 2.3.2 Statistical analysis

Antioxidant indices were analyzed using one-way ANOVA with SPSS 20.0 software, where *P*<0.05 was deemed statistically significant and the data results were expressed as “mean ± SE”. The Spearman rank correlation method was employed to evaluate the relationship between the microbiome and antioxidant indices, where correlations with |R|>0.5 were considered strong, and statistical significance was defined as *P*<0.05. A correlation heatmap was constructed using the “Complex Heatmap” package in R (R vegan package, 2.7-0).

## 3 Results

### 3.1 Effect of camel milk on antioxidant capacity of mouse liver

Differential analysis of liver antioxidant indices across four sample groups revealed the following (**Table 1**): TP concentrations in the CM and FCM groups were significantly higher than those in the DW group, and the FCM group was significantly higher than PFCM group (*P*<0.05); MDA activity in the CM, FCM, and PFCM groups was significantly reduced compared to the DW group, and FCM group was significantly reduced compared to CM group (*P*<0.05); SOD activity in the CM and PFCM groups was significantly lower than in the DW and FCM groups (*P*<0.05); TAC in the PFCM group was significantly higher than CM group (*P*<0.05).

**Table 1:** Comparison of antioxidant indices in liver of mice after camel milk treatment.

Items	DW group	CM group	FCM group	PFCM group
TP (mg/g)	15.54 ± 0.30^a^	17.28 ± 0.54^bc^	17.92 ± 0.59^c^	16.30 ± 0.57^ab^
CAT (mmol/min/g tissue)	10.94 ± 0.25	11.95 ± 0.38	12.07 ± 0.78	11.56 ± 0.34
MDA (nmol/g tissue)	3.27 ± 0.28^c^	2.19 ± 0.08^b^	1.50 ± 0.18^a^	1.92 ± 0.08^ab^
SOD (U/g tissue)	105.83 ± 8.92^b^	68.76 ± 6.32^a^	98.74 ± 15.52^b^	44.98 ± 3.29^a^
TAC (U/g tissue)	9.25 ± 1.87^ab^	6.36 ± 1.15^a^	6.76 ± 1.42^ab^	11.07 ± 1.28^b^
GSH-PX(U/g tissue)	0.37 ± 0.10	0.56 ± 0.12	0.68 ± 0.12	0.32 ± 0.12

Different uppercase letters denote significant differences at *P*<0.05.

### 3.2 Metagenomic analysis

Metagenomic analysis of the colon contents from 32 mice yielded 204,222.99M of Raw Data, comprising 1,361,486,694 Raw Reads. On average, each sample produced 6,381.97M of Raw Data and 42,546,459 Raw Reads. Following quality control, 204,079.86M of Clean Data was obtained, representing over 99% of the Raw Data. The GC content of the Clean Data exceeded 47%, with an average of 49.07%. Additionally, the Q20 and Q30 values for the Clean Data were above 96.5% and 91.3%, respectively (**Supplementary Table S4**).

**Figure 1:**
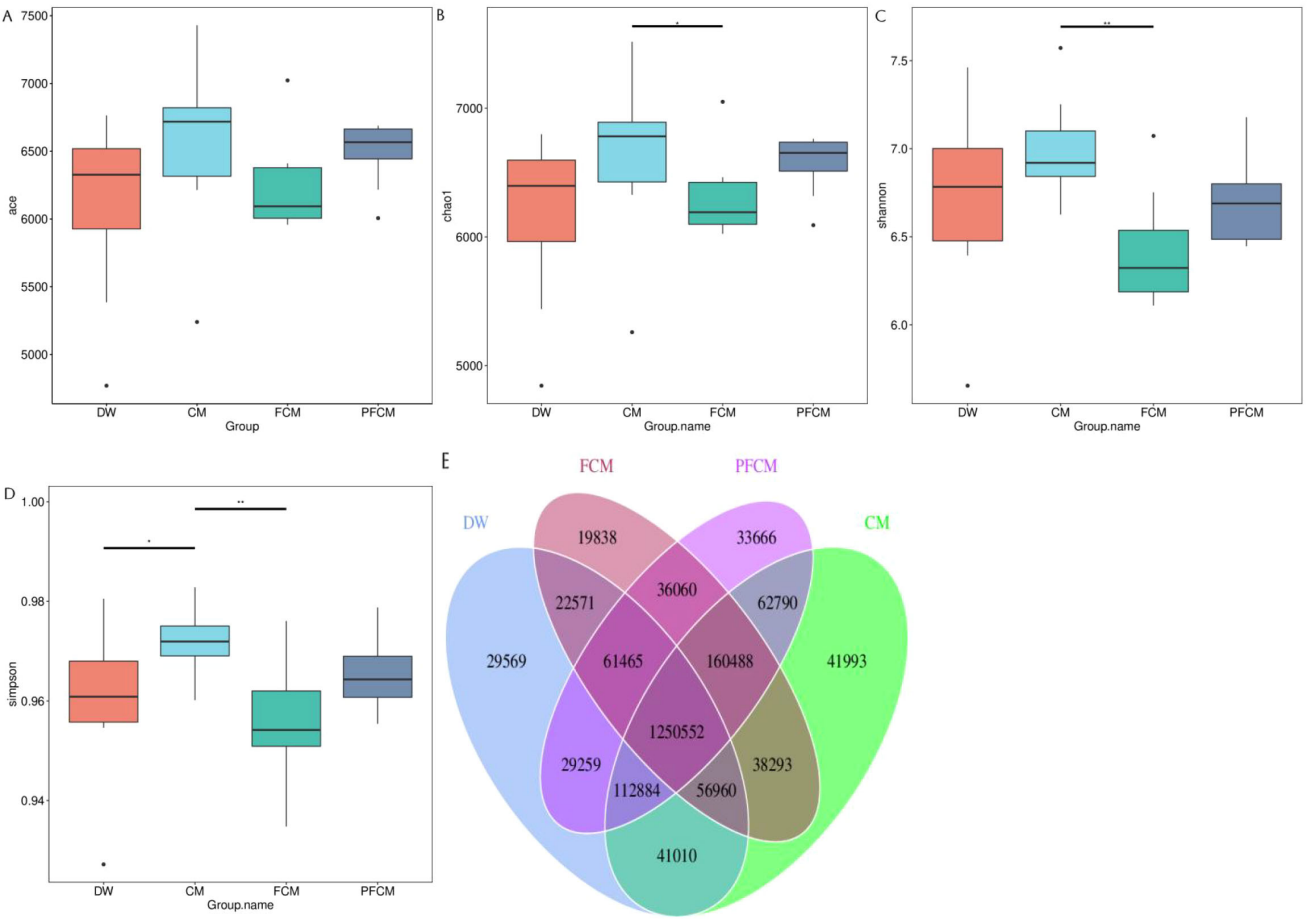
Diversity analysis of mouse gut microbiota. **(A)** ACE Index Box Plot. **(B)** Chao1 Index Box Plot. **(C)** Shannon Index Box Plot. **(D)** Simpson Index Box Plot. **(E)** Venn diagram of the gene number.

The intestinal microbiome across the four mouse groups was compared, with results depicted in **Figure 1**. Regarding α diversity (**Figures 1A–D**), the Simpson index in the CM group was significantly higher than in the DW group (*P*<0.05), while the Chao, Shannon, and Simpson indices of the CM group surpassed those of the FCM group (*P*<0.05). Venn diagram analysis revealed 1,250,552 common genes across all groups, with 29,569, 41,993, 19,838, and 33,666 genes uniquely identified in the DW, CM, FCM, and PFCM groups, respectively (**Figure 1E**).

### 3.3 Composition spectrum and taxonomic differences of mouse gut microbiota

The microbial composition of the four sample groups was analyzed using Clean Data. The dominant phyla included Bacteroidota (48.81 ± 8.74%), Bacillota (19.40 ± 7.37%), and Actinomycetota (3.17 ± 1.93%), which together comprised over 70% of the total microbial community. The most abundant genera were Lactobacillus (3.75 ± 2.71%), Faecalibaculum (1.25 ± 1.45%), Bacteroides (3.27 ± 1.24%), and Alistipes (3.50 ± 1.47%). The dominant species included Muribaculaceae bacterium (4.46 ± 1.19%), Muribaculaceae bacterium Isolate-104 (HZI) (4.22 ± 2.11%), Lactobacillus intestinalis (1.55 ± 1.32%), and Faecalibaculum rodentium (1.25 ± 1.45%).

**Supplementary Figure S1A** shows the relative abundance at the phylum level among the four groups, compared to the DW group, the relative abundance of Actinomycetota was higher in the CM group. The relative abundance of Bacteroidota was lower in the FCM group, while Bacillota, Actinomycetota, and Pseudomonadota increased. In the PFCM group, the relative abundance of Bacillota and Actinomycetota was higher. Compared with the FCM group, the relative abundance of Pseudomonadota decreased and the relative abundance of Uroviricota increased in the CM and PFCM groups. At the genus level, the CM group exhibited increase in Lactobacillus, Adlercreutzia, and Roseburia compared to the DW group (**Supplementary Figure S1B**). In the FCM group, Faecalibaculum and Adlercreutzia showed increases, while Odoribacter significantly. In the PFCM group, Lactobacillus, Adlercreutzia, and Limosilactobacillus were more abundant than those in the DW group. The relative abundance of Faecalibaculum in the FCM group was higher than that in the CM group and the PFCM group, and the relative abundance of Roseburia and Odoribacter was lower than that in the CM group. The relative abundance of Alistipes in the CM group was higher than that in the FCM group and the PFCM group. The relative abundance of Limosilactobacillus in the PFCM group was higher than that in the CM group and the FCM group.

A total of 4,732 microorganisms were identified across all samples (**Supplementary Figure S2**). Of these, 3,197 species were shared among all four groups, with the CM group exhibiting the highest species diversity (4,193 species) and the FCM group the lowest (3,935 species). Additionally, 259 species were exclusive to the DW group, 222 species to the CM group, 116 species to the FCM group, and 164 species to the PFCM group.

**Figure 2:**
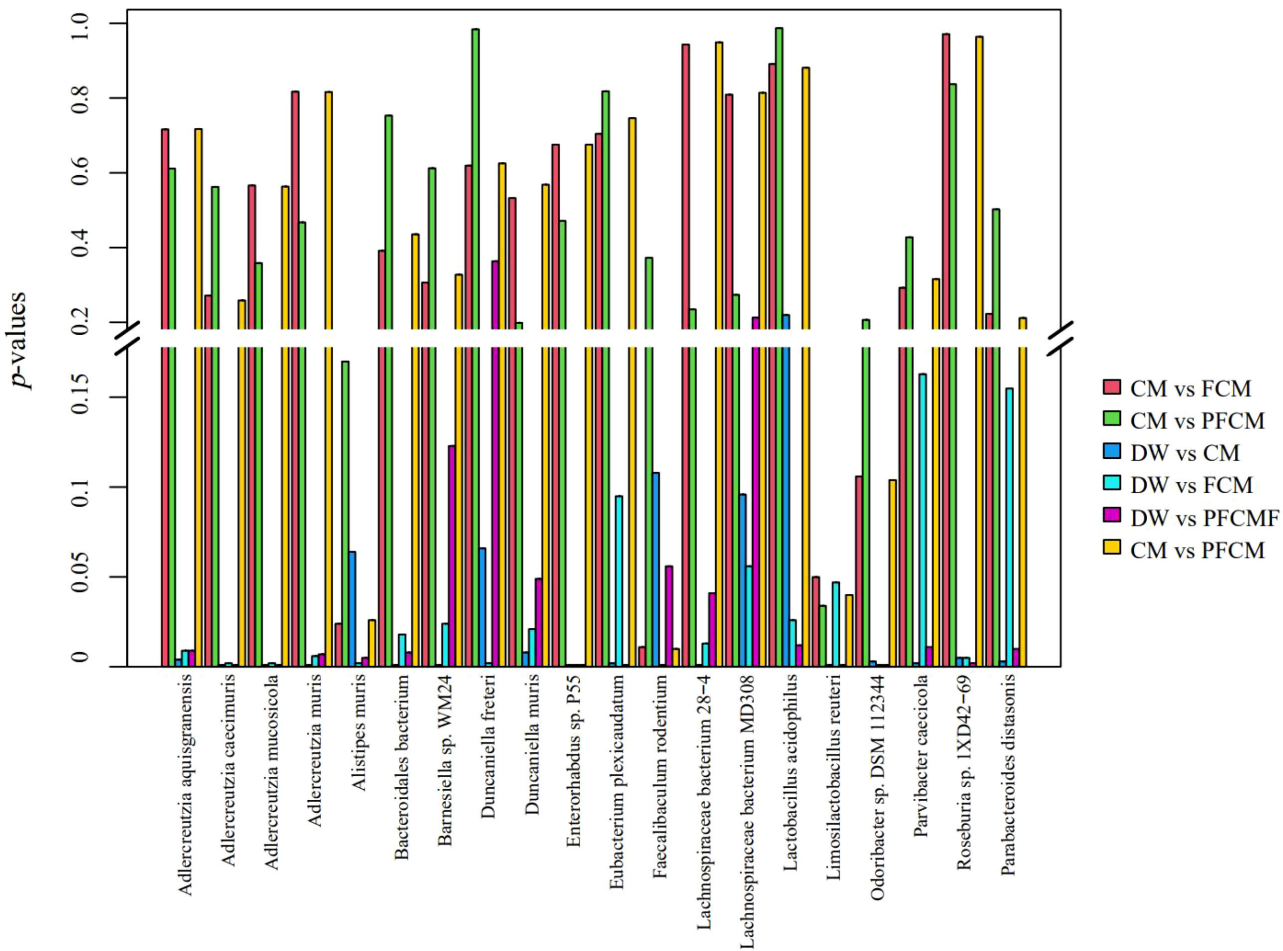
Species-level differences in gut microbiota among groups.

To examine microbial community diversity across groups, species-level differences were analyzed (**Figure 2**). The key differential species identified included Lactobacillus acidophilus, Faecalibaculum rodentium, Alistipes muris, Limosilactobacillus reuteri, Odoribacter sp. DSM 112344, Duncaniella freteri, Adlercreutzia caecimuris, Enterorhabdus sp. P55, Eubacterium plexicaudatum, Bacteroidales bacterium, Lachnospiraceae bacterium 28-4, Adlercreutzia muris, Adlercreutzia mucosicola, Parvibacter caecicola, Lachnospiraceae bacterium MD308, Duncaniella muris, Barnesiella sp. WM24, Roseburia sp. 1XD42-69, Adlercreutzia aquisgranensis, and Parabacteroides distasonis. Compared to the DW group, the CM group exhibited significantly higher abundances of Parvibacter caecicola, Adlercreutzia aquisgranensis, Adlercreutzia caecimuris, Adlercreutzia mucosicola, Adlercreutzia muris, Enterorhabdus sp. P55, Lactobacillus acidophilus, Roseburia sp. 1XD42-69, and Lachnospiraceae bacterium MD308, while the abundance of Duncaniella muris decreased (*P*< 0.05). In the FCM group, Adlercreutzia caecimuris, Adlercreutzia mucosicola, Enterorhabdus sp. P55, and Faecalibaculum rodentium showed significant increases, whereas Duncaniella freteri, Odoribacter sp. DSM 112344, and Alistipes muris were significantly reduced (*P*< 0.05). The PFCM group displayed notable increases in the abundance of Parvibacter caecicola, Adlercreutzia caecimuris, Adlercreutzia mucosicola, Adlercreutzia muris, Enterorhabdus sp. P55, Limosilactobacillus reuteri, Eubacterium plexicaudatum, Roseburia sp. 1XD42-69, and Lachnospiraceae bacterium 28-4, while Barnesiella sp. WM24, Parabacteroides distasonis, and Bacteroidales bacterium were significantly decreased (*P*< 0.05). Compared to the CM group, the FCM group exhibited significantly higher abundances of Faecalibaculum rodentium, while the abundance of Alistipes muris and Limosilactobacillus reuteri decreased (*P*< 0.05).

### 3.4 Correlation between species-level gut microbiota and antioxidant indices in mice

**Figure 3:**
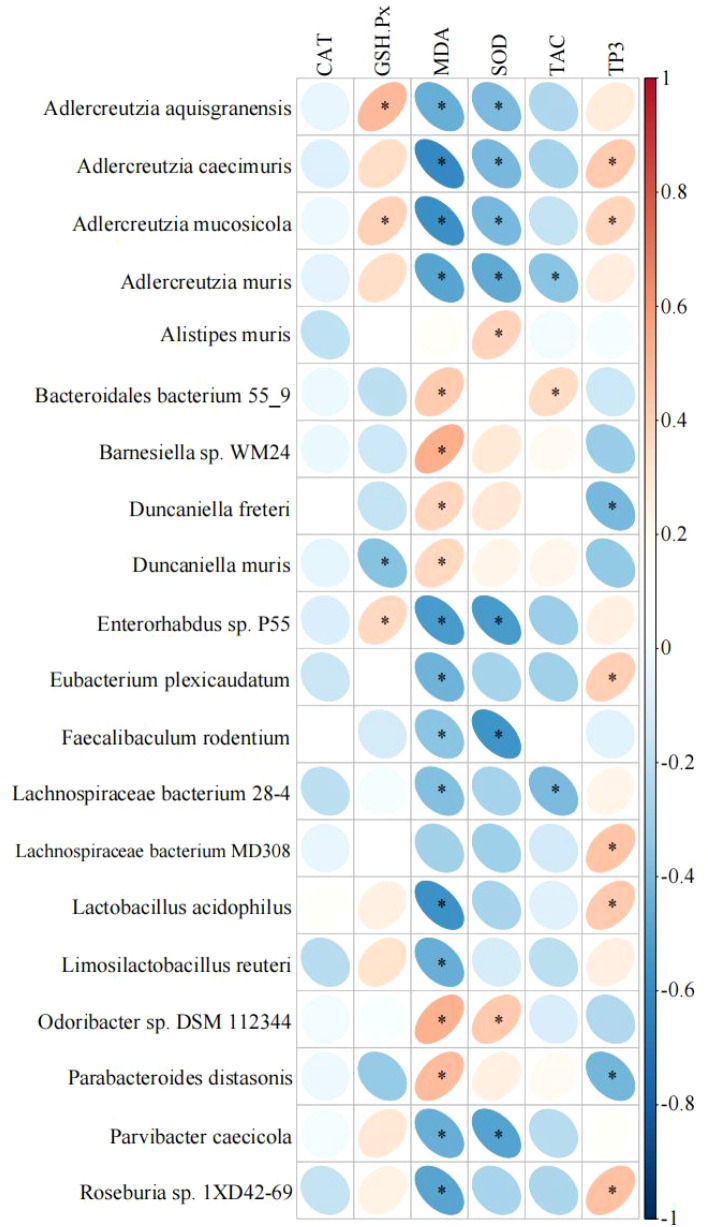
Correlation heatmap of antioxidant indices and microorganisms. *mean *P*< 0.05.

Spearman correlation analysis was conducted between antioxidant indices in mouse liver and the abundance of the most prominent microorganisms with species-specific differences (**Figure 3**). The data revealed a significant positive correlation between Adlercreutzia aquisgranensis, Adlercreutzia mucosicola, and Enterorhabdus sp. P55 with GSH-Px (*P*< 0.05), while Duncaniella muris exhibited a significant negative correlation with GSH-Px (*P*< 0.05). Barnesiella sp. WM24 and Odoribacter sp. DSM 112344 were positively correlated with MDA (*P*< 0.05), whereas Adlercreutzia caecimuris, Adlercreutzia mucosicola, Enterorhabdus sp. P55, and Lactobacillus acidophilus showed negative correlations with MDA (*P*< 0.05). A significant positive correlation between Odoribacter sp. DSM 112344 and SOD was observed (*P*< 0.05), while Adlercreutzia mucosicola, Adlercreutzia muris, Enterorhabdus sp. P55, Faecalibaculum rodentium, and Parvibacter caecicola were negatively correlated with SOD (*P*< 0.05). Bacteroidales bacterium 55-9 exhibited a positive correlation with TAC (*P*< 0.05), while Adlercreutzia muris and Lachnospiraceae bacterium 28-4 were negatively correlated with TAC (*P*< 0.05). Finally, Adlercreutzia caecimuris, Eubacterium plexicaudatum, Lachnospiraceae bacterium MD308, Lactobacillus acidophilus, and Roseburia sp. 1XD42-69 demonstrated significant positive correlations with TP (*P*< 0.05), whereas Parabacteroides distasonis showed a significant negative correlation with TP (*P*< 0.05).

### 3.5 Functional annotation analysis

The KEGG database annotation results revealed that Metabolism represented the most annotated category at level 1, while Organismal Systems ranked the lowest, as illustrated in **Supplementary Figures S3A, B**. At level 2, six out of the top ten most abundant pathways were associated with metabolism, as shown in **Supplementary Figures S3B**. The annotated genes were distributed in the following order, from most to least: carbohydrate metabolism, amino acid metabolism, metabolism of cofactors and vitamins, translation, membrane transport, energy metabolism, glycan biosynthesis and metabolism, nucleotide metabolism, replication and repair, and signal transduction.

**Figure 4:**
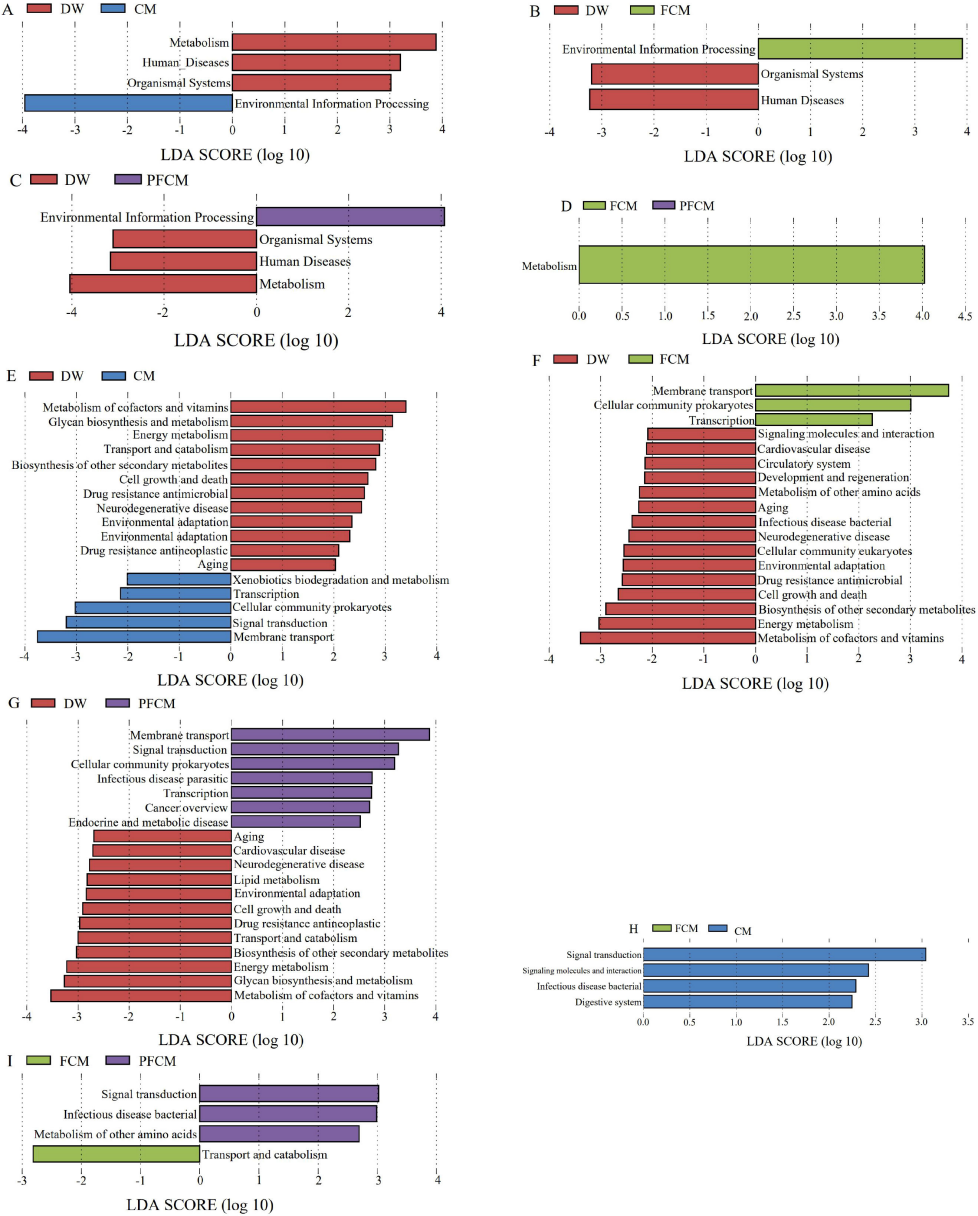
Distribution of LDA values of differential functions. **(A)** LDA value distribution diagram of differential function between CM group VS DW group at level 1. **(B)** LDA value distribution diagram of differential function between FCM group VS DW group at level 1. **(C)** LDA value distribution diagram of differential function between PFCM group VS DW group at level 1. **(D)** LDA value distribution diagram of differential function between FCM group VS PFCM group at level 1. **(E)** LDA value distribution diagram of differential function between CM group VS DW group at level 2. **(F)** LDA value distribution diagram of differential function between FCM group VS DW group at level 2. **(G)** LDA value distribution diagram of differential function between PFCM group VS DW group at level 2. **(H)** LDA value distribution diagram of differential function between FCM group VS CM group at level 2. **(I)** LDA value distribution diagram of differential function between FCM group VS PFCM group at level 2.

LEfSe analysis of differential functions among the groups was conducted with reference to the KEGG database. The results (**Figure 4**; **Supplementary Figure S4**) revealed that, at level 1, the Human Disease and Organismal Systems pathways were significantly upregulated in the camel milk treatment groups compared to the DW group, while the Environmental Information Processing pathway was downregulated (*P*< 0.05). Additionally, the Metabolism pathway showed significant upregulation in both the CM and PFCM groups (*P*< 0.05). Metabolism pathway showed significant upregulation in FCM group compared to the PFCM group (*P*< 0.05). At level 2, the Xenobiotics biodegradation and metabolism, Transcription, Cellularcommunity prokaryotes, Signal transduction, Membrane transport pathways were notably upregulated in the CM group, whereas pathways related to Metabolism of cofactors and vitamins, Glycan biosynthesis and metabolism, Energy metabolism, Transport and catabolism, Biosynthesis of other secondary metabolites, Cell growth and death, Drug resistance antimicrobial, Neurodegenerative disease, Cardiovascular disease, Environmental adaptation, Drug resistanceantineoplastic, and Aging were significantly downregulated (*P*< 0.05). In the FCM group, Membrane Transport, Cellular Community Prokaryotes, and Transcription were upregulated, while pathways including Signaling Molecules and Interaction, Cardiovascular Disease, Circulatory System, Development and Regeneration, Metabolism of Other Amino Acids, Aging, Infectious Disease Bacterial, Neurodegenerative Disease, Cellular Community Eukaryotes, Environmental Adaptation, Drug Resistance Antimicrobial, Cell Growth and Death, Biosynthesis of Other Secondary Metabolites, Energy Metabolism, and Metabolism of Cofactors and Vitamins were significantly downregulated (*P*< 0.05). In the PFCM group, Membrane Transport, Signal Transduction, Cellular Community Prokaryotes, Infectious Disease Parasitic, Transcription, Cancer Overview, and Endocrine and Metabolic Diseases were significantly upregulated, while pathways related to Aging, Cardiovascular Disease, Neurodegenerative Disease, Lipid Metabolism, Environmental Adaptation, Cell Growth and Death, Drug Resistance Antineoplastic, Transport and Catabolism, Biosynthesis of Other Secondary Metabolites, Energy Metabolism, Glycan Biosynthesis and Metabolism, and Metabolism of Cofactors and Vitamins were downregulated (*P*< 0.05). Signal transduction, Signaling molecules and interaction, Infectious disease bacterial, Digestive system were notably upregulated in the CM group compared to the PFCM group (*P*< 0.05). Compared to the FCM group, in the PFCM group, Signal transduction, Infectious disease bacterial, Metabolism of other amino acids were upregulated, and Transport and catabolism was downregulated. Compared to the CM group, the FCM group exhibited significantly higher abundances of Faecalibaculum rodentium, while the abundance of Alistipes muris and Limosilactobacillus reuteri decreased (*P*< 0.05).

## 4 Discussion

The immune system generates reactive oxygen species (ROS) to eliminate pathogens. To preserve cellular integrity, ROS levels are tightly changed by the antioxidant system. However, under internal or external stress, this balance may be disrupted, leading to an imbalance between ROS production and the antioxidant defense system, resulting in cellular, tissue, and organ damage (Harrison et al., 2011; Sadasivam et al., 2022). The antioxidant system consists of both enzymatic antioxidants, such as SOD and CAT, and non-enzymatic antioxidants, like GSH (Bhor et al., 2004), which catalyze reactions involved in ROS metabolism. By neutralizing ROS generated during metabolic processes, the system maintains a dynamic equilibrium between radical production and elimination, thus contributing significantly to the body’s defense mechanisms (Valko et al., 2006). The liver, with its high oxygen consumption, is a key organ in metabolic processes, making the maintenance of oxidative balance within it essential (Fang et al., 2018). H_2_O_2_, a major ROS, is converted to water by CAT (Schieber and Chandel, 2014), while SOD catalyzes the conversion of superoxide into oxygen and H_2_O_2_. In response to stimuli, SOD is rapidly induced, and CAT neutralizes H_2_O_2_ by decomposing it into molecular oxygen and water (He et al., 2017). MDA, a marker of lipid peroxidation, serves as an indicator of cellular antioxidant activity (Jelic et al., 2021). The study results indicated that after the ingestion of camel milk with various treatments, liver SOD activity in mice decreased, suggesting a reduction in H_2_O_2_ production and, consequently, alleviated liver oxidative stress. This may be due to feedback inhibition due to reduced oxidative stress (Lodhi et al., 2021). Our results also showed that the CAT concentration of mice in different treatment groups was higher than that in the DW group. CAT can decompose H_2_O_2_ into H_2_O and O_2_, thereby eliminating the activity of H_2_O_2_ and protecting liver cells from oxidative damage (Schieber and Chandel, 2014). This indicates that different treatments of camel milk can reduce oxidative stress in the liver. In our study, the FCM group had the highest CAT concentration, which showed that FCM had the best anti-oxidative stress effect, but there was no significant difference between all groups, which may be related to the small sample size of our study. An increase in GSH-Px activity was noted in the CM and FCM groups, reflecting enhanced liver antioxidant capacity. MDA, a lipid peroxide generated by the attack of oxygen free radicals on unsaturated fatty acids, serves as an indicator of cellular oxidative damage and correlates with the activity of endogenous antioxidants. The results of this study showed that after consuming camel milk with different treatments, the MDA activity in the liver of mice was significantly reduced, especially in the FCM group, which was significantly lower than that in the CM group and the PFCM group, indicating that the antioxidant capacity of the mouse liver was effectively improved (Zeng et al., 2020). Therefore, compared with the DW group, the intake of camel milk with different treatments can and relieve oxidative stress of the mouse liver, and fermented camel milk is more effective in alleviating oxidative stress. In addition, although we used mice of different sexes in our study, we did not conduct separate analyses due to the small sample size, so we were unable to evaluate the effect of sex.

A balanced microbiome is essential for overall health, and dysbiosis, characterized by an imbalance between commensal bacteria and pathogenic microorganisms, is implicated in various diseases (Halfvarson et al., 2017; James et al., 2020). Research results show that lactose intake increases the growth of lactobacilli and bifidobacteria (Yuan et al., 2022). In addition, components in the milk fat globule membrane on the surface of milk fat have a bactericidal effect on microorganisms such as Campylobacter jejuni and Salmonella enteritidis *in vitro (* Ye et al., 2021). Other studies have also shown that the abundance of Lactobacillus, Bifidobacterium, and Akkermansia in the feces of infants fed fermented goat milk is higher (Yuan et al., 2022). This shows that the intake of milk and dairy products will have a regulatory effect on intestinal microorganisms. This study analyzed the gene count and α diversity of the mouse gut microbiota. Both the ACE and Chao indexes were higher in the CM group compared to the DW group, aligning with findings from related studies (Hao et al., 2022). Oligosaccharides and antibacterial components in camel milk may be the reason why camel milk changes the gastrointestinal microbial diversity of mice (He et al., 2022). Additionally, the CM group exhibited the largest number of unique genes, while the FCM group had the fewest, further corroborating previous research (Schloss et al., 2009). These results suggest notable differences in microbiota composition across the groups.

Studies have shown that the intestinal microorganisms of rats fed with milk are mainly Actinobacteriota, Acidobacteriota, Bacteroidota, Verrucomicrobiota, and Firmicutes, and the abundance of Bacteroidota increased by 15% after 3 months of feeding (Wang et al., 2024). Bacteroidota is the most abundant in the intestinal flora of mice fed with camel milk fermentation (Aljutaily et al., 2025). The microorganisms in the feces of mice fed with camel milk are mainly Bacteroidota, Bacillota, and Actinomycetota, which is consistent with previous research results. In addition, the relative abundance of Firmicutes and Bacteroidota in mice fed with milk decreased (Cakebread et al., 2022). In our study, we also found that the Bacteroidota in the FCM group of mice was significantly lower than that in the DW group. This may be because Bacteroidota can utilize carbohydrates. After oral administration of FCM, the type and composition of polysaccharides changed, resulting in changes in the microbial community (Sonnenburg et al., 2010). After consuming fermented dairy products, the abundance of Lactobacillus, Bifidobacterium and Blautia in the intestines of mice increased (Chen et al., 2024b). After feeding with cow’s milk, the abundance of Bacteroides (mainly Bacteroides uniformis), Blautia (mainly Blautia producta) and Roseburia (mainly Roseburia faecis) in the intestines of mice increased (Mukarromah et al., 2025). The main microorganisms found at the species level in this study included Adlercreutzia mucosicola, Adlercreutzia muris, Lactobacillus acidophilus and Enterorhabdus sp. P55, which rarely appeared in other studies related to dairy products, and their regulatory mechanisms need further study.

Bacteroidota was the most abundant phylum across all groups, suggesting its potential role in metabolism (Parkar et al., 2013). In the FCM group, its abundance significantly decreased, consistent with previous studies (Machate et al., 2020), implying that this phylum may contribute to reduced oxidative capacity via metabolic pathways. Research results show that Bacteroidales can produce immunomodulatory capsular polysaccharide A, thereby enhancing the body’s immune function (Ramakrishna et al., 2019). In addition, its main metabolites in *in vitro* culture are amino acids and derivatives, nucleotides and organic acids, which may also affect the body’s antioxidant capacity (Fernandez-Cantos et al., 2024). Our study found that Bacteroidales bacterium was significantly correlated with MDA and TAC in liver tissue, indicating that Bacteroidales bacterium may affect MDA and TAC through metabolites, especially organic acids. Actinomycetota, known for its involvement in carbohydrate metabolism and the degradation of amino acids and fatty acids (Lugli et al., 2017), also produces secondary metabolites with antioxidant properties (Singh and Dubey, 2020). These metabolites, such as butyrate, exert antioxidant effects by modulating immune cell function and reducing ROS generation (Chen et al., 2024a; Zhang et al., 2024). In this study, the abundance of Actinomycetota in the intestinal flora of all groups of mice was increased after consuming camel milk, which is consistent with the research results of dromedary camel milk (Sheikh et al., 2022). In addition, some studies have shown that supplementing the culture medium with free radical scavengers (SOD) can promote the growth of Actinomycetota (Takahashi, 2024), indicating that the antioxidant activity of camel milk may be related to its metabolites.

The extracellular polysaccharide of Adlercreutzia exhibits SOD-like activity, effectively inhibiting α-glucosidase, enhancing tyrosinase activity, and increasing polyphenol content, thereby demonstrating potent antioxidant properties (Bajpai et al., 2024). In the present study, increased abundance of Adlercreutzia was observed following camel milk consumption under various treatments, potentially contributing to the milk’s antioxidant effects. Adlercreutzia mucosicola, a species within the genus, shares antioxidant properties typical of the genus. The relative abundance of Adlercreutzia mucosicola was significantly higher in all treatment groups compared to the DW group. Correlation analysis revealed positive associations with GSH-Px and negative associations with MDA and SOD. The antioxidant activity of MDA and SOD is primarily linked to H_2_O_2_ (Ayranci et al., 2019), suggesting that these bacteria may enhance antioxidant capacity via pathways involving carbohydrate and amino acid metabolism. Furthermore, Adlercreutzia muris showed negative correlations with SOD and TCA activity, with its relative abundance notably increasing in the CM and PFCM groups, indicating its potential role in regulating camel milk’s antioxidant capacity. Adlercreutzia is negatively correlated with bile acid, dehydrosaffynol, and arctiopicrin (Liu CH. et al., 2024), and the intestinal microbiota and bile acid interact with each other and may jointly promote intestinal inflammation under dysbiosis (Ke et al., 2020). This shows that Adlercreutzia mucosicola may change the antioxidant capacity of mice by regulating bile acid, but because we did not detect metabolites, this part needs further confirmation. This study conducted an enrichment analysis of the KEGG metabolic pathway and found that camel milk can change the metabolism of mice, indicating that Adlercreutzia mucosicola may achieve this function through metabolic pathways.

Lactobacillus can facilitate the hydrolysis of macromolecular proteins, such as casein, into peptides or amino acids. Its antioxidant properties are primarily mediated through enzyme production and fermentation. For instance, fermentation enhances its ability to scavenge and reduce 1,1-diphenyl-2-picrylhydrazyl free radicals, thus boosting antioxidant capacity (Hu et al., 2020). In this study, Lactobacillus abundance increased in both CM and PFCM groups, suggesting that these treatments may enhance antioxidant capacity via Lactobacillus fermentation. Studies have shown that supplementing with Lactobacillus can increase the level of antioxidant enzymes and enhance the body’s antioxidant capacity, thereby reducing the production of MDA produced by lipid peroxidation (Wang et al., 2025). In this study, Lactobacillus acidophilus was negatively correlated with the MDA activity of mice, and its abundance was significantly increased in each group, indicating that Lactobacillus acidophilus may change the antioxidant activity of mice by regulating lipid metabolism. This species can colonize the gut, providing various protective benefits (Li et al., 2019; Hu et al., 2020). Existing studies have demonstrated its role in regulating intestinal permeability (Martoni et al., 2020), suggesting that its antioxidant effects may be linked to organic systems and cofactor and vitamin metabolism. Additionally, Lactobacillaceae and Lactobacillus species are associated with short-chain fatty acid production (Zhao et al., 2012). Short-chain fatty acids have been identified as regulators of Nrf2-Keap1 signaling (Kong et al., 2021), influencing histone deacetylase inhibition and/or Nrf2 nuclear translocation, thereby promoting antioxidant activity (Zou et al., 2021; Kallie et al., 2024). The increased abundance of Lactobacillus acidophilus further supports its role in antioxidant effects through metabolic pathways.

Although we analyzed the effects of different camel milk treatments on the antioxidant capacity of mice from a microbial perspective, we did not conduct a comparative analysis with cow’s milk or human milk, and the number of mice tested was small, which may affect the persuasiveness of our results.

## 5 Conclusions

This study examines the impact of various camel milk treatments on liver antioxidant indices and the composition and function of gut microbiota in mice. We found that the PFCM group had the highest total antioxidant capacity and the lowest SOD content, and the MDA activity of the CM, FCM and PFCM groups was lower than that of the DW group. This indicates that camel milk can change the antioxidant capacity of mouse liver, thereby alleviating the body’s oxidative stress. In addition, the intestinal microbial structure of mice changed after feeding camel milk, especially compared with the DW group, the relative abundance of Adlercreutzia caecimuris, Adlercreutzia mucosicola, and Enterorhabdus sp. P55 in the CM group, FCM group, and PFCM group increased significantly. In addition, the relative abundance of Faecalibaculum rodentium in the FCM group was significantly higher than that in the CM group. Among them, Adlercreutzia mucosicola, Adlercreutzia muris, Lactobacillus acidophilus, and Enterorhabdus sp. P55 were related to the antioxidant indexes of mice. These microorganisms may change the antioxidant capacity of mice through pathways such as Carbohydrate metabolism, Amino acid metabolism, Membrane transport, and Signal transduction, thereby alleviating the body’s oxidative stress. The findings offer valuable insights for the development of functional camel milk products.

## Data Availability

The data presented in the study are deposited in the NCBI repository, accession number PRJNA1232340.

## Supplementary material

Supplementary Figure 1 Effects of camel milk on mouse gut microbiota composition (p: Phylum; g: Genus). **(A)** The relative abundance at the phylum level. **(B)** The relative abundance at the Genus level.

Supplementary Figure 2 Effects of camel milk on species levels in mouse gut microbiota compositions

Supplementary Figure 3 Statistics of the number of genes annotated.Functional annotation. **(A)** Relative abundance bar graph at level 1, **(B)** Relative abundance bar graph at level 2; Level 1 corresponds to the first tier of KEGG metabolic pathways, including six major metabolic pathways. Level 2 represents the second tier, consisting of 57 seed pathways within KEGG metabolism

Supplementary Figure 4 LEfSe analysis of differential functions among the groups.

Supplementary Table 1 Nutritional components of camel milk with different treatments.

Supplementary Table 2 Fatty acid content in each group

Supplementary Table 3 Mouse weight information

Supplementary Table 4 Sequencing results.
