# Supplementary material for: Changes in antioxidant capacity and gut microbiota in mice after intake of camel milk
Source: Front Cell Infect Microbiol. 2025 Sep 3;15:1621031. doi: 10.3389/fcimb.2025.1621031 (PMC12440968; doi:10.3389/fcimb.2025.1621031)
Supplement: Supplementary Figure 1 — Effects of camel milk on mouse gut microbiota composition (p: Phylum; g: Genus). (A) The relative abundance at the phylum level. (B) The relative abundance at the Genus level. [file Supplementaryfile1.doc]

Supplementary Material

# Supplementary Figures and Tables

## Supplementary Tables

# Table S1 Nutritional components of camel milk with different treatments.

| Nutritional composition | Protein（%） | Fat（%） | Lactose（%） |
| --- | --- | --- | --- |
| CM | 3.32 | 4.32 | 5.23 |
| FCM | 2.91 | 4.33 | 4.49 |

# Table S2 Fatty acid content in each group

| Fatty acid | CM (ng/mL) | FCM (ng/mL) | PFCM (ng/mL) |
| --- | --- | --- | --- |
| Caprylic acid | 7187.04 | 12407.86 | 9744.84 |
| Decanoic acid | 1525.44 | 2113.24 | 1450.25 |
| Hendecanoic acid | 319.61 | 345.67 | 278.16 |
| Dodecanoic acid | 20536.98 | 24170.88 | 15734.71 |
| Tridecanoic acid | 2240.56 | 3139.85 | 2234.93 |
| Tetradecanoic acid | 183906.56 | 228475.32 | 140485.72 |
| Myristoleic acid | 32864.62 | 44033.45 | 29576.88 |
| Pentadecanoic acid | 21306.67 | 27641.59 | 21148.83 |
| trans-10-Pentadecenoic acid | 2117.76 | 3143.82 | 1943.74 |
| Hexadecanoic acid | 263030.54 | 316335.01 | 203308.92 |
| Palmitoleic acid | 230123.49 | 318586.43 | 205815.23 |
| Palmitelaidic acid | 9501.28 | 14223.17 | 11172.37 |
| Heptadecanoic acid | 8458.36 | 10801.24 | 6453.09 |
| cis-10-Heptadecenoic acid | 13551.15 | 18544.23 | 12543.41 |
| trans-10-Heptadecenoic acid | 1756.09 | 2932.45 | 2454.61 |
| Octadecanoic acid | 219179.43 | 257171.28 | 160504.83 |
| Oleic acid | 29363.69 | 36108.34 | 23610.01 |
| Petroselaidic acid | 338766.99 | 417104.37 | 229319.87 |
| trans-Vaccenic acid | 14969.06 | 20516.23 | 21861.62 |
| Linoleic acid | 77136.56 | 187681.34 | 118513.42 |
| Linoelaidic acid | 6514.15 | 9661.88 | 8176.93 |
| Arachidic acid | 5281.87 | 5392.91 | 3257.31 |
| gamma-Linolenic acid | 5614.19 | 8718.11 | 7225.98 |
| cis-11-Eicosenoic acid | 1209.73 | 1273.39 | 896.87 |
| trans-11-Eicosenoic acid | 7453.13 | 10058.69 | 6291.94 |
| alpha-Linolenic acid | 45751.96 | 56042.55 | 31772.18 |
| Heneicosanoic acid | 668.66 | 573.76 | 323.48 |
| cis-11,14-Eicosadienoic acid | 3966.45 | 4889.37 | 4021.01 |
| Docosanoic acid | 1058.29 | 1094.04 | 728.11 |
| homo-gamma-Linolenic acid | 5741.48 | 13094.46 | 7389.38 |
| Erucic acid | 239.26 | 278.31 | 152.76 |
| Brassidic acid | 390.94 | 367.96 | 289.077 |
| cis-11,14,17-Eicosatrienoic acid | 5192.75 | 9335.37 | 7444.56 |
| Arachidonic acid | 11550.19 | 49518.63 | 34784.52 |
| cis-13,16-Docosadienoic acid | 250.11 | 235.45 | 198.76 |
| cis-5,8,11,14,17-Eicosapentaenoic acid | 4356.63 | 14819.82 | 11495.20 |
| cis-7,10,13,16-Docosic acidtraenoic acid | 2608.85 | 5816.81 | 3797.96 |
| cis-7,10,13,16,19-Docosapentaenoic acid | 343.86 | 967.85 | 680.85 |
| cis-4,7,10,13,16-Docosapentaenoic acid | 4647.62 | 17749.86 | 13779.54 |
| Tetracosanoic acid | 716.74 | 789.92 | 422.23 |
| Nervonic acid | 205.52 | 222.54 | 164.20 |
| cis-4,7,10,13,16,19-Docosahexaenoic acid | 1029.78 | 3041.29 | 2006.01 |

# Table S3 Mouse weight information.

| Day | 1^st^ day | 8^th^ day | 15^th^ day | 22^nd^ day | 29^th^ day |
| --- | --- | --- | --- | --- | --- |
| DW (g) | 23.69±0.46 | 26.63±0.91 | 30.08±1.47 | 31.17±1.66 | 31.92±1.44 |
| CM(g) | 23.13±0.34 | 26.81±0.57 | 30.05±0.81 | 30.98±0.79 | 32.63±0.96 |
| FCM(g) | 23.74±0.13 | 26.62±0.43 | 29.93±0.68 | 30.76±0.78 | 31.26±0.92 |
| PFCM(g) | 23.39±0.29 | 26.40±0.41 | 29.98±0.85 | 30.18±1.05 | 31.85±0.89 |

Data is presented in mean ± SD format.

# Table S4 Sequencing results.

| Sample | Raw Data（M） | Raw Reads | Clean Data（M） | Clean　Q20 | Clean　Q30 | Clean　GC(%) | Effective(%) |
| --- | --- | --- | --- | --- | --- | --- | --- |
| DW1 | 6284.03 | 41893530 | 6280.39 | 98.01 | 94.27 | 48.34 | 99.942 |
| DW2 | 6816.63 | 45444186 | 6811.44 | 97.83 | 93.97 | 47.13 | 99.924 |
| DW3 | 6773.58 | 45157210 | 6769.15 | 97.16 | 92.41 | 48.61 | 99.935 |
| DW4 | 6639.70 | 44264674 | 6635.68 | 97.84 | 93.84 | 49.69 | 99.939 |
| DW5 | 6561.48 | 43743208 | 6557.39 | 97.53 | 93.19 | 50.23 | 99.938 |
| DW6 | 6209.20 | 41394696 | 6201.42 | 97.86 | 94 | 48.94 | 99.875 |
| DW7 | 5992.54 | 39950234 | 5990.17 | 97.94 | 94.1 | 49.97 | 99.961 |
| DW8 | 6205.35 | 41369028 | 6201.41 | 98.06 | 94.45 | 49.64 | 99.936 |
| CM1 | 6314.01 | 42093380 | 6309.90 | 97.62 | 93.44 | 48.23 | 99.935 |
| CM2 | 6548.04 | 43653568 | 6544.60 | 97.1 | 92.3 | 50.34 | 99.948 |
| CM3 | 6519.16 | 43461064 | 6515.38 | 97.52 | 93.12 | 48.18 | 99.942 |
| CM4 | 6446.75 | 42978324 | 6444.20 | 97.53 | 93.25 | 48.7 | 99.96 |
| CM5 | 5981.31 | 39875406 | 5977.92 | 97.7 | 93.62 | 49.79 | 99.943 |
| CM6 | 6757.50 | 45050010 | 6755.28 | 97.67 | 93.56 | 49.64 | 99.967 |
| CM7 | 6407.03 | 42713566 | 6399.78 | 97.71 | 93.58 | 49.39 | 99.887 |
| CM8 | 6346.16 | 42307732 | 6343.53 | 97.51 | 93.16 | 49.84 | 99.959 |
| FCM1 | 6088.23 | 40588174 | 6085.08 | 98.08 | 94.39 | 50.7 | 99.948 |
| FCM2 | 6236.64 | 41577618 | 6233.61 | 97.58 | 93.29 | 48.78 | 99.951 |
| FCM3 | 6518.87 | 43459150 | 6515.61 | 97.72 | 93.68 | 48.53 | 99.95 |
| FCM4 | 6321.87 | 42145806 | 6318.99 | 97.67 | 93.5 | 48.14 | 99.954 |
| FCM5 | 6427.40 | 42849348 | 6423.79 | 97.37 | 92.87 | 49.72 | 99.944 |
| FCM6 | 6244.32 | 41628770 | 6242.23 | 97.42 | 92.99 | 50.5 | 99.967 |
| FCM7 | 6170.26 | 41135060 | 6167.83 | 97.69 | 93.65 | 50.15 | 99.961 |
| FCM8 | 6304.03 | 42026860 | 6294.73 | 97.15 | 92.4 | 47.09 | 99.852 |
| PFCM1 | 6292.59 | 41950618 | 6288.19 | 97.52 | 93.17 | 47.94 | 99.93 |
| PFCM2 | 6402.23 | 42681560 | 6399.32 | 97.59 | 93.37 | 48.72 | 99.954 |
| PFCM3 | 6269.29 | 41795294 | 6263.99 | 97.5 | 93.11 | 47.89 | 99.915 |
| PFCM4 | 6407.73 | 42718180 | 6404.76 | 97.14 | 92.37 | 49.31 | 99.954 |
| PFCM5 | 6415.01 | 42766712 | 6410.51 | 97.28 | 92.73 | 49.23 | 99.93 |
| PFCM6 | 6507.17 | 43381154 | 6504.49 | 97.52 | 93.17 | 48.78 | 99.959 |
| PFCM7 | 6455.89 | 43039288 | 6452.41 | 97 | 92.05 | 48.66 | 99.946 |
| PFCM8 | 6358.99 | 42393286 | 6336.68 | 96.5 | 91.38 | 49.28 | 99.649 |

## Supplementary Figures


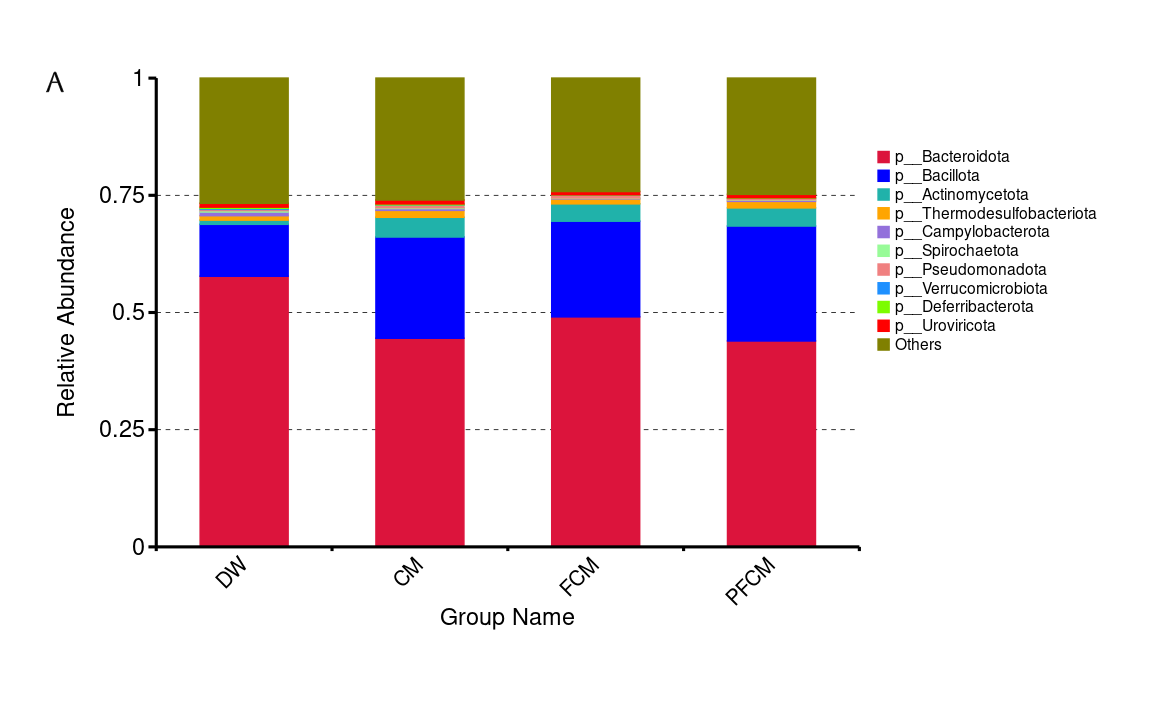

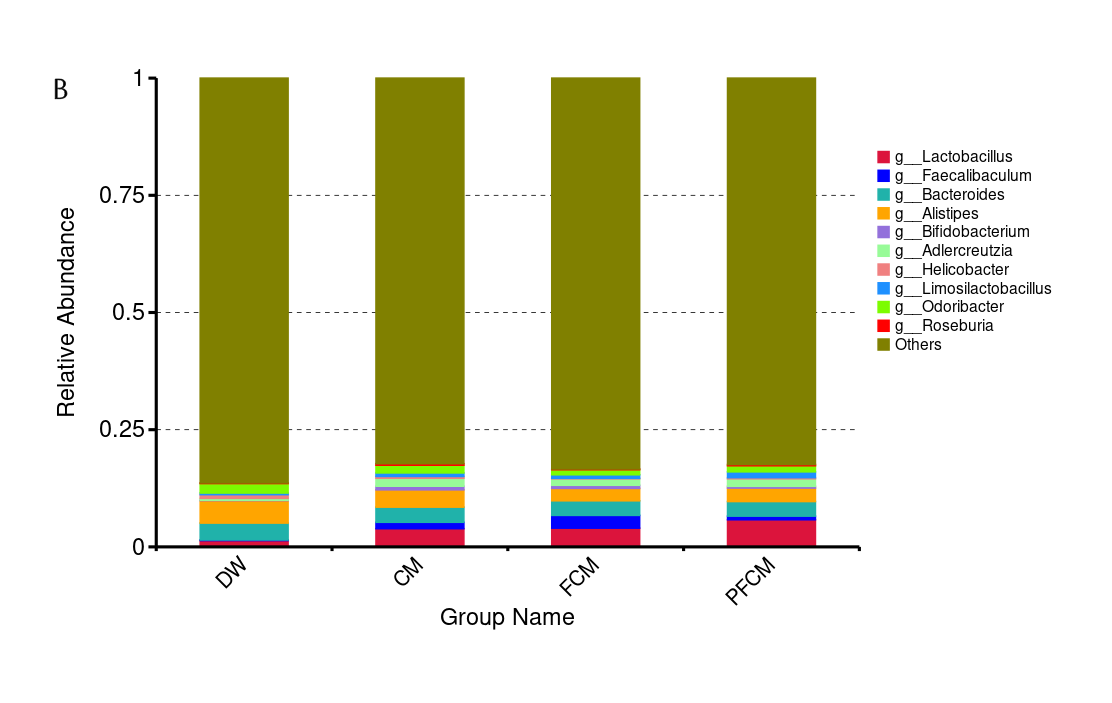


FIGURE S1 Effects of camel milk on mouse gut microbiota composition (p: Phylum; g: Genus). (A) The relative abundance at the phylum level. (B) The relative abundance at the Genus level.


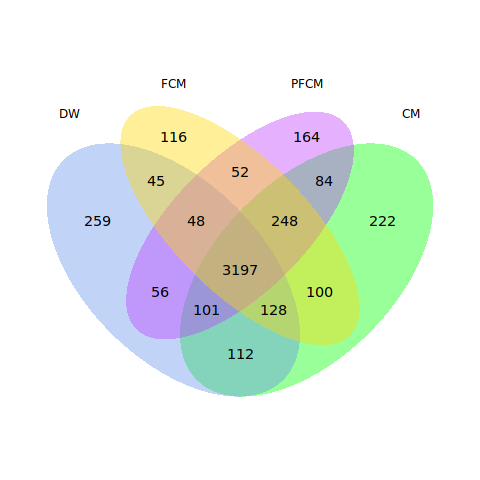


FIGURE S2 Effects of camel milk on species levels in mouse gut microbiota compositions


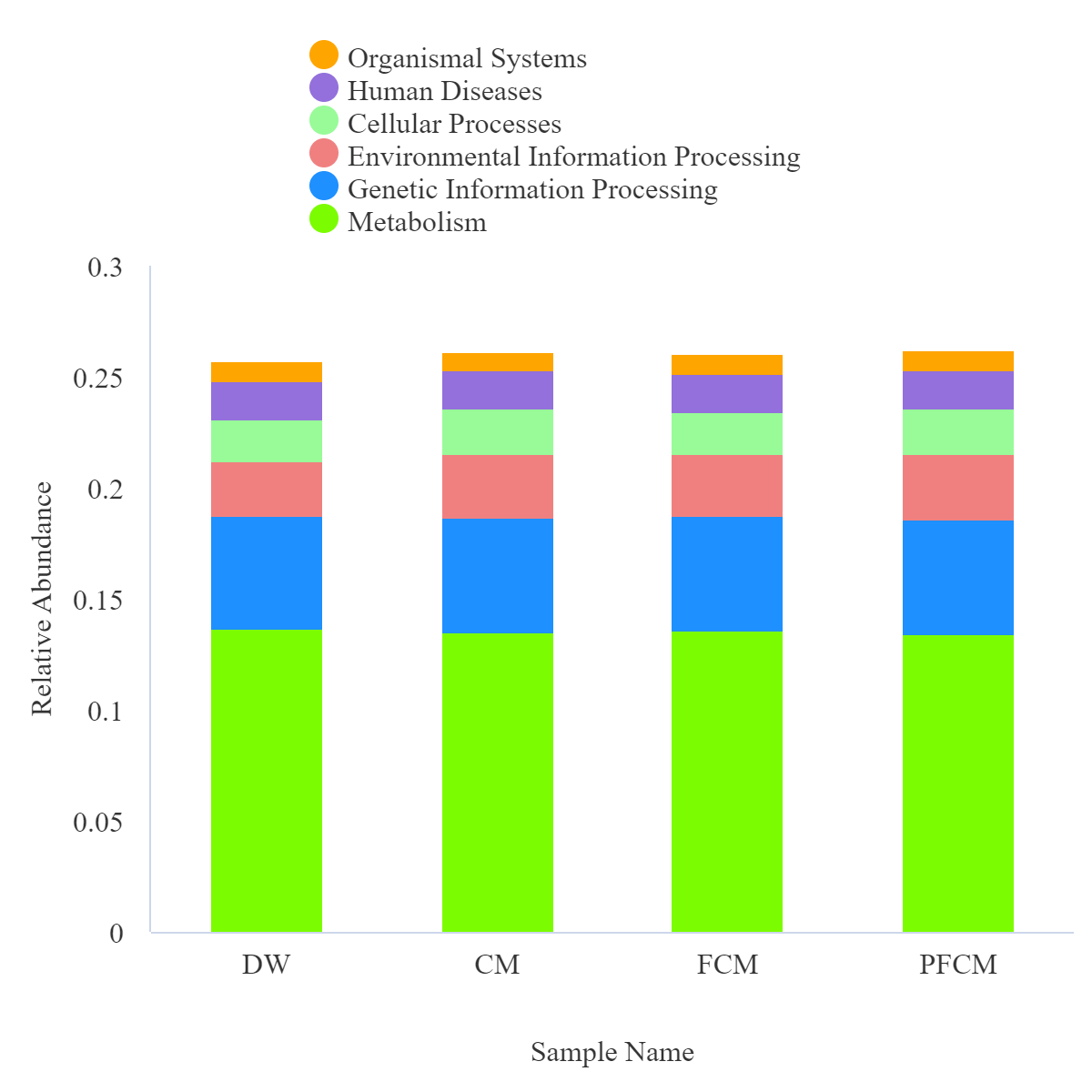

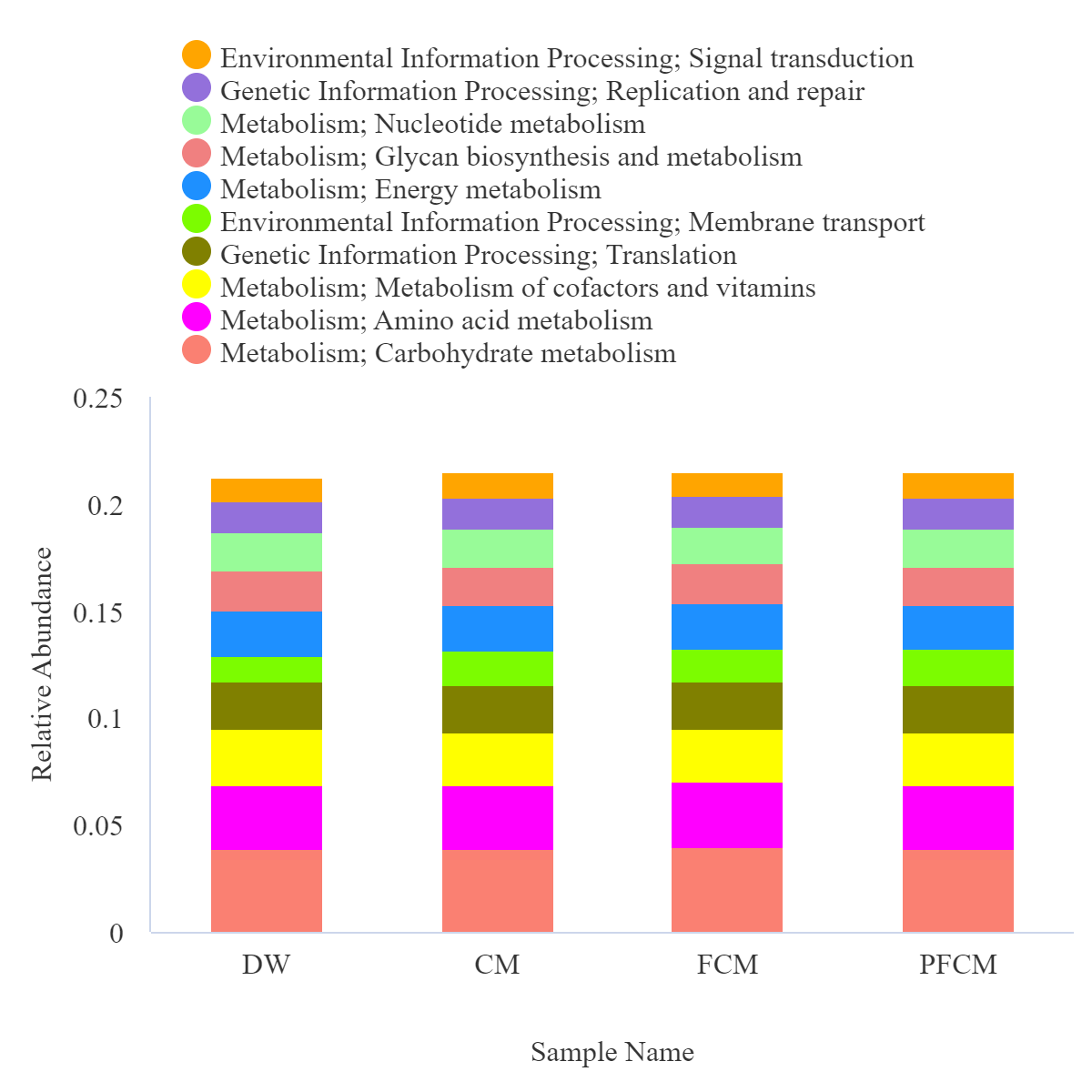


FIGURE S3 Statistics of the number of genes annotated.Functional annotation. (A) Relative abundance bar graph at level 1, (B) Relative abundance bar graph at level 2; Level 1 corresponds to the first tier of KEGG metabolic pathways, including six major metabolic pathways. Level 2 represents the second tier, consisting of 57 seed pathways within KEGG metabolism


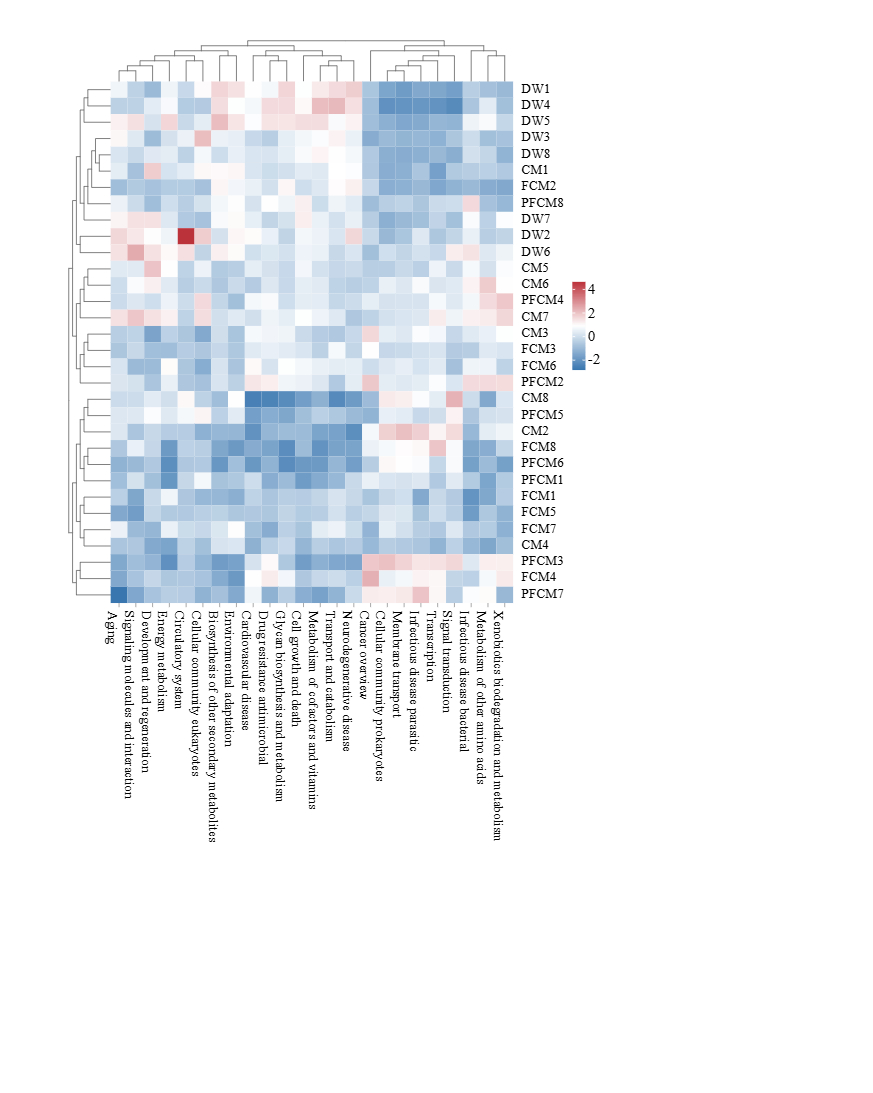


FIGURE S4 LEfSe analysis of differential functions among the groups
